# IS*6110* Copy Number in Multi-Host *Mycobacterium bovis* Strains Circulating in Bovine Tuberculosis Endemic French Regions

**DOI:** 10.3389/fmicb.2022.891902

**Published:** 2022-06-23

**Authors:** Ciriac Charles, Cyril Conde, Franck Biet, Maria Laura Boschiroli, Lorraine Michelet

**Affiliations:** ^1^ANSES, Animal Health Laboratory, National Reference Laboratory for Tuberculosis, Paris-Est University, Paris, France; ^2^INRAE, ISP, Université de Tours, Nouzilly, France

**Keywords:** *Mycobacterium bovis*, bovine tuberculosis (bTB), insertion sequence (IS), IS*6110*, France

## Abstract

IS*6110* is an insertion sequence found in the *Mycobacterium tuberculosis* complex, to which *Mycobacterium bovis* belongs, which can play a role in genome plasticity and in bacterial evolution. In this study, the abundance and location of IS*6110* on *M. bovis* genomic data of French animal field strains were studied. A first analysis was performed on a panel of 81 strains that reflect the national *M. bovis* population’s genetic diversity. The results show that more than one-third of them are IS*6110* multicopy and that 10% have IS*6110* in a high copy number (more than 6 copies). Multicopy strains are those circulating in the regions where prevalence was above the national average. Further study of 93 such strains, with an IS*6110* copy number of 10-12, showed stability of IS*6110* copy number and genome location over time and between host species. The correlation between *M. bovis* multicopy strains and high bovine tuberculosis (bTB) prevalence leads us to consider whether their epidemiological success could be partly due to genetic changes originated by IS*6110* transposition.

## Introduction

IS*6110* is a specific insertion sequence of the *Mycobacterium tuberculosis* complex (MTBC) historically used for genotyping techniques, mainly restriction fragment length polymorphism (RFLP) IS*6110*, the former gold standard to identify epidemiologically linked isolates of the MTBC species (van Soolingen et al., 1994). Easier and more discriminatory methods such as spoligotyping, mycobacterial interspersed repetitive unit-variable number tandem repeat (MIRU-VNTR), and more recently, whole-genome single nucleotide polymorphism (wgSNP) have been developed (Kamerbeek et al., 1997; Supply et al., 2006; Comas et al., 2009; Driscoll, 2009; Hauer et al., 2019).

IS*6110* is of particular interest due to its role in genome plasticity and bacterial evolution. In fact, this insertion sequence can promote gene inactivation and structural variation (insertion, inversion, or deletion) and can act as a mobile promoter (Soto et al., 2004; Alonso et al., 2013). As an example, the insertion of IS*6110* in the *phoP* promoter has been described as the cause of gene upregulation and virulence increase of the *Mycobacterium bovis* (*M. bovis*) B strain, which caused severe human multidrug resistant tuberculosis outbreaks in Spain (Samper et al., 1997; Soto et al., 2004). In addition, the correlation between the high amount of IS*6110* in *M. tuberculosis* Beijing lineage and its increased virulence, antibiotic resistance, and the ability of these strains to better adapt to the environment have been described (Kremer et al., 2004; McEvoy et al., 2007; Merker et al., 2013). The number of IS*6110* copies varies among MTBC members: strains belonging to the *M. tuberculosis* Beijing lineage have the most important number of IS*6110* copies (15 on average), while *M. bovis* strains present only one or few copies of IS*6110* (Gonzalo-Asensio et al., 2018). Nonetheless, the recent publication of a new complete genome sequence of a French field strain, *M. bovis* Mb3601, showed that this particular strain possess 11 IS*6110* copies (Branger et al., 2020). This strain, which belongs to the European 3 (Eu3) complex clonal, is found in an endemic bovine tuberculosis (bTB) region (Côte d’Or in Burgundy, Central-East France) and belongs to one of the most abundant genotypes in France in the last years (Hauer et al., 2015).

Bovine tuberculosis is an old worldwide chronic zoonotic disease due to *M. bovis*. The main maintenance host species of *M. bovis* is domestic cattle but this bacterium can circulate in multi-host systems that include not only domestic but also wild animals and their environment, therefore, explaining the persistence of the pathogen in some areas (Réveillaud et al., 2018). France obtained the officially bTB free (OTF) status in 2001 (Benet et al., 2006). However, this OTF status is threatened by a steady rise of bTB cattle outbreaks over the past 15 years, partly due to more efficient surveillance in cattle and also due to the presence of wildlife infection which contributes to the persistence of the disease at regional levels.

A previous study shows a strong regionalization of bTB in France caused by specific *M. bovis* genotypes in different endemic regions (Hauer et al., 2015). Totally, 80% of bTB outbreaks in France in the last years are due to 3 major *M. bovis* genotypes (Delavenne et al., 2021): SB0120-DHV genotype (SB0120-VNTR 5 3 5 3 9 4 5 6) found in Dordogne and Haute-Vienne departments (Nouvelle Aquitaine region); SB0120-CO genotype (SB0120-VNTR 5 5 4 3 11 4 5 6) found in Côte d’Or department (Burgundy region); Cluster A/F4 family genotypes (Hauer et al., 2019), especially due to SB0821/F007 and SB0832/F015 genotypes in the Atlantic Pyrenees department (Nouvelle Aquitaine region) and SB0840/F001 genotype in Corsica.

The persistence of these main genotypes could be due to the ability of the strains to adapt to the environment. In fact, these strains seem to be maintained and expand geographically despite the implementation of control measures adapted to each context (Boschiroli et al., 2015; Hauer et al., 2015; Delavenne et al., 2021).

The objectives of this study were (i) to establish using a whole-genome sequencing (WGS) approach the abundance of IS*6110* copy and their chromosomal distribution in 80 strains representative of French *M. bovis* genotypes diversity and (ii) to evaluate the level of the IS*6110* sequence stability and their orthology in a panel of 92 sympatric strains of SB0120-CO genotype evolving in a multi-host system.

## Materials and Methods

### Genomes of *Mycobacterium bovis* French Strains

The first panel (panel 1) constituted genomes of 80 *M. bovis* strains isolated from French bovine tuberculosis outbreaks between 1983 and 2011 which were selected from a previous study (Supplementary Figure 1 and Supplementary Table 1) (Hauer et al., 2019). DNA from bacterial clones was extracted using the phenol-chloroform method detailed in a previous study and sequenced with Illumina HiSeq technology [paired-end (2 × 100 bp)] (Hauer et al., 2019). The second panel (panel 2) constituted 92 SB0120-CO strains that have been selected from another study (Michelet et al., 2018). Bacterial thermolysates without any further purification steps were used as DNA sources. DNA sequencing was carried out with Illumina HiSeq technology [paired-end (2 × 250 bp)] (Michelet et al., 2018). These strains, isolated from the bTB endemic region in Côte d’Or (Central East France), were obtained from different animal hosts in the framework of the French bTB control campaign between 2009 and 2014 (Supplementary Figure 1 and Supplementary Table 1).

The short-read quality was evaluated using FastQC (version 0.11.9 with default parameters), and reads were trimmed with Sickle^1^ (version 1.33 with default parameters) using a quality phred-score of Q20 (Xia et al., 2016; Wingett and Andrews, 2018). The SPAdes (version 3.15.2 with careful option) tool was used to assemble short reads (Bankevich et al., 2012), and Prokka (version 1.14.6 with default parameters) was used on the genomes’ assemblies (Seemann, 2014) to check the absence of contamination of the short-read sequencing. Strains of panel 2 with more than 3 rRNA or with a size between 4,200,000 and 4,600,000 base pairs were considered for further analyses.

### Phylogenetic Analysis

Single nucleotide polymorphisms were obtained using the Bionumerics software, version 7.6 (AppliedMath, Belgium). Identified SNPs were selected according to strict criteria of the wgSNP module as follows: they had to be present on at least five reads in both forward and reverse directions, twelve base pairs had to separate them, they were not present in repetitive regions of the genome, and ambiguous SNPs [at least one unreliable (N) base, ambiguous (non-ATCG) base, or gap] were not included. The evolutionary trees were inferred on Mega (Kumar et al., 2016) using the maximum likelihood method (Hasegawa–Kishino–Yano model) based on concatenated and validated SNPs (panel 1, 8,981 SNPs for 81 genomes; panel 2, 124 SNPs for 93 genomes). The trees were drawn to scale, with branch lengths measured in the number of substitutions per site.

### IS*6110* Distribution Analysis

ISMapper (version 2.0.1) pipeline (Hawkey et al., 2015) was carried out in this study for IS*6110* identification on genome short-read sequences using Mb3601 reference genome (Branger et al., 2020). The employed script is available on GitHub.^2^ To achieve 100% detection with high confidence, average genome-wide read depths of < 75 × were excluded for panel 2 strains (Hawkey et al., 2015). Only 93 genomes (with Mb3601 reference strains) out of the initial 147 passed the quality selection described in this and the previous section.

For uncertain IS*6110* presence and/or localization (37 and 9 uncertain insertion sites in panel 1 and panel 2, respectively) due to low read depth in the IS insertion site, IS*6110* presence was manually checked with Integrative Genomics Viewer (Robinson et al., 2011) using the bam file generated by ISMapper.

### Orthology Analysis

Genomic positions were deduced through ISMapper analysis. Surrounding genes (other than IS*6110*) of these insertion sites were identified based on Mb3601 reference to determine orthologous genomic sites of the IS. Insertion sites present in the same coding sequence (CDS) are grouped in a common locus.

### IS*6110* Site Gene Ontology Enrichment Analysis

Gene ontology (GO) analysis was performed on the upstream and downstream genes of IS*6110* using their protein sequence. Functional annotation was performed using eggnog 5.0 (Huerta-Cepas et al., 2019). Specific colors were applied to the gene according to their Cluster of Orthologous Gene (COG) functional categories.

## Results

### IS*6110* Abundance in *Mycobacterium bovis* French Diversity

**FIGURE 1:**
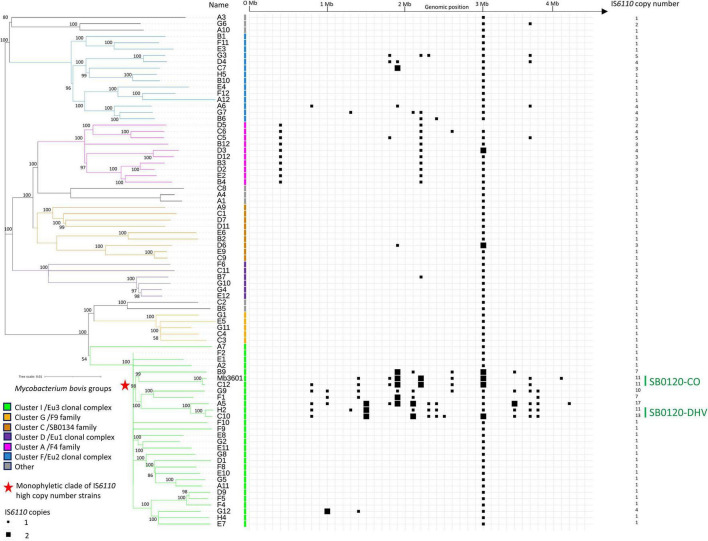
IS*6110* occurrence in *Mycobacterium bovis* French genetic diversity. Heatmap showing the presence and genomic position of IS*6110* in 81 genomes (80 genomes of *Mycobacterium bovis* representing French diversity and Mb3601 reference strains). The tree is based on 8,981 whole-genome single nucleotide polymorphism. The strains are grouped in 7 clusters that have been previously defined (Hauer et al., 2019).

Among the 81 *M. bovis* strains representing the French genotypic diversity of panel 1, 65% (53/81) have only one IS*6110* copy, 25% (20/81) are IS*6110* low copy number (2-5 IS*6110* copies), and 10% (8/81) present a high copy number with more than six copies (Figure 1 and Supplementary Table 1) (Fomukong et al., 1998; Gonzalo-Asensio et al., 2018). Among the different clusters described by Hauer and collaborators (Hauer et al., 2019), strains of Cluster C (SB0134) and those of the European 1 clonal complex (Smith et al., 2011) are mostly single copies, except for one strain in each cluster with two and three copies (B7 and D6, respectively). F9 family/cluster G strains are all IS*6110* single copies. The IS*6110* copy number seems to be correlated with the phylogenetic group (cluster or subcluster). All strains in Cluster A/F4 family group are IS*6110* multicopy with three common insertions, which are present in most of the strains belonging to this cluster (8/10). Among the 14 strains that belong to the European 2 (Eu2) clonal complex (Rodriguez-Campos et al., 2012), 43% of them (6/14), which are localized on two specific branches of the phylogenetic tree, possess more than one IS*6110* copy (3–5 copies). Notably, 31% of Eu3 clonal complex strains (9/29), including the Mb3601, are IS*6110* multicopy. This group includes the strain with the highest IS*6110* copy number, i.e., 16 copies, strain A5. Interestingly, these high copy number strains (7–16 copies) are all present in a specific monophyletic clade (8/8). Strains that have caused the majority of outbreaks in France in the last 10 years belong to strain groups, which are multicopy, with a low copy number of 2–5 copies for Cluster A/F4 family and a high copy number of 11–13 copies for SB0120-DHV and SB0120-CO.

In the 81 strains of panel 1, 58 insertion sites have been identified (insertion sites 1 to 58 related to position on the genome Mb3601) and are summarized in Supplementary Table 2. Almost 50% of these insertion sites (28/58) are only observed in a unique strain. Almost all these strains possess an IS*6110* insertion in the Direct Repeat (DR) locus (position 43). However, this insertion is absent in two Cluster A/F4 family strains: D5, with an SB0840 spoligotype and D12 with a SB0928 spoligotype. Strain D12 has an IS*6110* insertion in position 42, also observed in strain D3 which is phylogenetically close to strain D12. In addition to the number of copies, which seems to be related to certain strain groups, the localization of these insertions also seems to be conserved. For instance, Cluster A/F4 family strains have 3 common IS*6110* insertion sites (insertion sites 1, 33, and 43). Eight of these insertion sites are shared between several *M. bovis* groups. For example, insertion site 33 is common between Cluster A/F4 family (10 strains) and SB0120-CO genotype (2 strains), and insertion sites 19 and 50 are both shared by 7 strains of three different clonal groups.

SB0120-DHV genotype strains (strains H2 and C10) and the SB0120-CO genotype (strains C12 and Mb3601) strains have each 10 IS*6110* conserved insertion sites, but only two of them (insertion sites 43 and 50) are common to the four strains. Strain A5, phylogenetically closely to SB0120-DHV, has also these ten IS*6110* insertion sites but also shares a common insertion site (insertion site 19) with strains belonging to the monophyletic clade of IS*6110* high multicopy strains (SB0120-CO, F1, and B9 strains).

### Longitudinal Study of IS*6110* in Sympatric Strains

**FIGURE 2:**
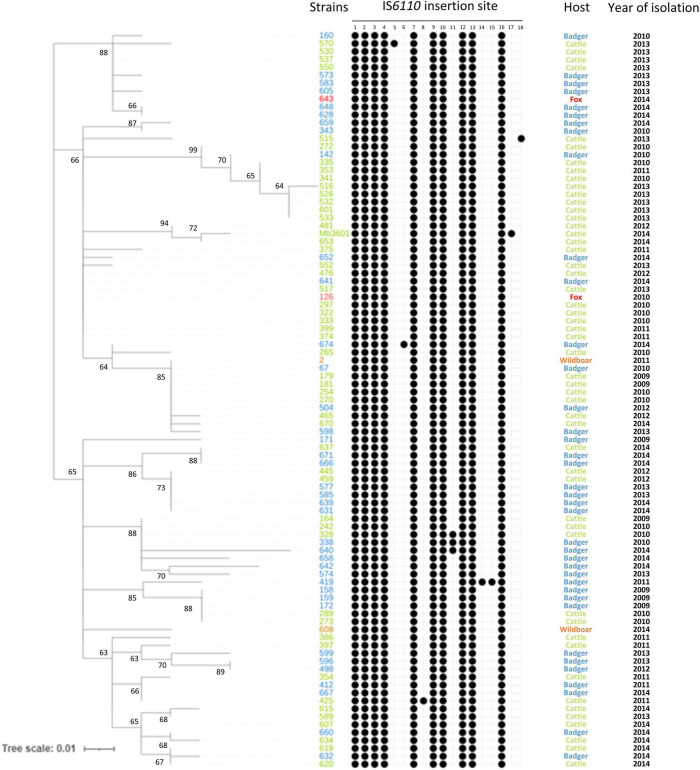
IS*6110* in *M. bovis* SB0120-CO over time and between different host species. Heatmap showing the presence or absence of IS*6110* in 93 sympatric SB0120-CO genomes. Black spots show the presence of IS*6110* in a specific site. Nucleotide positions of each IS*6110* site are described in Supplementary Table 3. The tree is based on 124 whole-genome single nucleotide polymorphism.

To further study the IS*6110* high copy number phenomenon in *M. bovis* strains, IS*6110* sites were searched in a panel of multicopy strains of the SB0120-CO genotype. The studied panel, panel 2, is composed of strains isolated over a period of 6 years and from four different species [cattle, badger (*Meles meles*), wild boar (*Sus scrofa*), and fox (*Vulpes vulpes*)]. SB0120-CO strains possess between 10 and 12 IS*6110* copies (Figure 2 and Supplementary Table 3). These insertion sites are stable over time; in fact, ten of them are found in all SB0120-CO strains of panel 1 and panel 2 (Figures 1, 2). Furthermore, IS*6110* genomic positions are also conserved independently of the animal species from which the strains were isolated. Eight other IS*6110* genomic positions exist but are unique to a specific strain. One of them is present in 3 very close SB0120-CO strains. Besides, SB0120-CO strains with an additional insertion are present on different branches of the tree. These insertions are neither recurrent in our sampling nor conserved over time or correlated to host adaptation.

### Genetic Impact of the IS*6110* Insertions

The analysis of IS*6110* genomic positions in the *M. bovis* French diversity (panel 1) revealed 58 insertion loci and shows that 62% of them are intragenic (36/58) (Supplementary Table 2). Of the 36 intragenic insertions, 7 are present in Mb3601, and their identities are confirmed with blast alignment on AF2122/97 reference genome (data not shown). However, the proportion of intragenic IS*6110* sites is different in several groups according to different IS*6110* copy numbers.

A schematic representation of insertion sites (Supplementary Table 4) shows that some IS*6110* insertion sites can be localized in different positions and orientations in the same insertion region such as in the Clustered Regularly Interspaced Short Palindromic Repeats CRISPR-associated (CRISPR-Cas) region, near the MBS3601_RS09105 locus or near the MBS3601_RS05055 locus.

The intragenic insertions are mostly found in Cluster A/F4 family, Cluster F/Eu2 clonal complex, and Cluster I/Eu3 clonal complex strains which can be explained by the presence of several copies of IS*6110* in these groups (Supplementary Table 2). Analyses of the genes interrupted by IS*6110* in the 3 most representative French *M. bovis* types defined previously, i.e., SB0120-CO, SB0120-DHV, and Cluster A/F4 family, show genes associated with 10 gene ontology pathways. Most genes interrupted by IS*6110* in Cluster A/F4 family and SB0120-CO strains have unknown, and “Replication, recombination and repair” functions but also “Coenzyme transport and metabolism,” “Defense mechanisms,” “Energy production and conversion,” and “Cell wall/membrane/envelope biogenesis” function in SB0120-CO. These functions are not found in SB0120-DHV, where genes involved in “Cell motility” and “Transcription” functions are those presenting the most interruptions. These insertion sites in specific genes can be conserved in the same *M. bovis* cluster such as *rpfD*, *moeY*, and *cas1* for SB0120-CO, MB3601_RS02050 (hyaluronidase/chondrosulfatase) for Cluster A/F4 family, or MBS3601_RS11780 (*LysR* family transcriptional regulator) and MBS3601_RS16315 (PPE family protein) for SB0120-DHV. Some other genes can be interrupted in several *M. bovis* clusters such as *radD* (12 strains from 2 different clusters) or *plcD* (7 strains from 3 different clusters). However, further study on these insertion sites shows the difference in genomic position and/or in IS orientation among *M. bovis* clusters (Supplementary Tables 2, 4).

**FIGURE 3:**
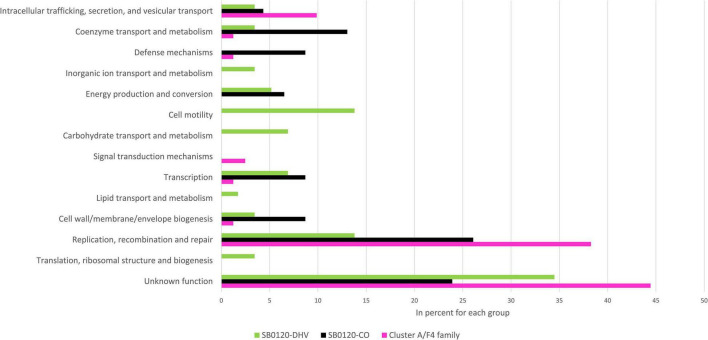
Gene-environment of IS*6110* sites in *M. bovis* French diversity. Gene Ontology (GO) of coding sequences (CDSs) near IS*6110* sites in 3 *M. bovis* groups (SB0120-DHV in green, SB0120-CO in black, and Cluster A/F4 family in pink).

A total of 13 GO pathways present in upstream or downstream of IS*6110* insertion loci in SB0120-CO, SB0120-DHV, and Cluster A/F4 family could be highlighted (Figure 3). Most of these genes are associated with an unknown function GO category. The “Replication, recombination and repair” GO category is the most frequently represented category with a determined function in the three groups, with 38% (31/81) in Cluster A/F4 family, 26% (12/46) in SB0120-CO, and 14% (8/58) SB0120-DHV.

## Discussion

*Mycobacterium bovis* strains are commonly considered as possessing one or few copies of IS*6110* (Gonzalo-Asensio et al., 2018), and only few strains were described as IS*6110* multicopy (Soto et al., 2004; Allix et al., 2006; Gonzalo-Asensio et al., 2018; Branger et al., 2020). However, our study focusing on French *M. bovis* diversity shows that more than one-third of our samples are multicopy. Specifically, strains of Cluster A/F4 family, SB0120-CO and SB0120-DHV genotypes, which are those circulating in the most bTB prevalent regions in the past 10 years (Delavenne et al., 2021), are IS*6110* multicopy. This observation leads us to consider whether IS*6110* transposition could be partly responsible for their epidemiological success, although other explanations could support this success, notably the drop in strain diversity (Boschiroli et al., 2015; Hauer et al., 2015; Delavenne et al., 2021) and the associated bottleneck following the establishment of control programs. Moreover, other genetic events can also contribute to the success of these strains (indel, SNP). Further analyses are needed to combine SNP and deletion events as well as IS*6110* transposition events.

Strains with the highest IS*6110* copy numbers belong to the Eu3 clonal complex and in particular to a specific monophyletic clade in the phylogenetic tree to which the two monophyletic types SB0120-CO and SB0120-DHV belong. *In silico* results describing the presence of IS*6110* high copy number strains in the same monophyletic clade are sustained by the description of the 11 IS*6110* copies in the Mb3601 complete genome (Branger et al., 2020) and that of strain F1 (7 IS*6110* copies). F1 is characterized by spoligotype SB0162 (Supplementary Table 1) and found not only in France but also in Belgium and previously described by IS*6110* RFLP analysis as presenting 8–11 copies, which is close to our results (Allix et al., 2006).

According to the results on SB0120-CO sympatric strains, IS*6110* copy number and insertion site seem to be stable over time among this type of strain, with an average of 10 copies and recurrence in their genomic positions. Furthermore, a specific host adaptation through IS*6110*-linked changes does not seem to have taken place as suggested for highly epidemic *M. tuberculosis* strains (Beijing lineage) (Gonzalo-Asensio et al., 2018), given that no strain variability is observed, neither on the copy number nor in IS*6110* insertion site, independently of the different animal hosts from which the strains were isolated from. This high stability over time and independently of the affected animal species demonstrates that *M. bovis* strains do not seem to evolve *via* IS*6110* transposition for specific host adaptation as suggested by Gonzalo-Asensio et al. (2018). Both this stability and independence of the animal host were also confirmed in IS*6110* multicopy SB0120-DHV and Cluster A/F4 family strains of SB0821 and SB0832 spoligotypes, and also in SB0134 single-copy strains infecting livestock and wildlife in several regions in France (data not shown). Furthermore, the stability of the 3 common IS*6110* sites in Cluster A/F4 family strains suggests the evolution of these strains from a common ancestor. However, if the genotypes SB0120-CO and SB0120-DHV have each 10 conserved insertion sites, only 2 are common between the two types. As expected, one of them is present in the DR locus (insertion 43). The second one (insertion 50) is present in an IS*6110* Preferential Locus (ipl) region which is described to be a hotspot of IS*6110* (Fang and Forbes, 1997; Roychowdhury et al., 2015). In fact, this insertion is also present in strains of the other groups such as Cluster A/F4 family and Eu2 clonal complex.

As expected, almost all strains in our panel (79 out of 81) have the IS*6110* in the DR locus. This insertion site is common to most MTBC species and is defined as the ancestral insertion of a common ancestor (Hermans et al., 1990; Dale, 1995; Philipp et al., 1996; Thorne et al., 2011; Gonzalo-Asensio et al., 2018). MTBC that lacks the DR IS*6110* has previously been described (Refrégier et al., 2020) and may be explained by recombination events between two IS*6110* and IS transpositions (Gonzalo-Asensio et al., 2018; Shitikov et al., 2019; Refrégier et al., 2020).

The specific insertion in several genes such as *moeY* in SB0120-CO strains or *radD* in Cluster A/F4 family and SB0120-DHV strains shows that their expression is not essential for bacterial virulence and transmission given that these genotypes are prevalent in France (Delavenne et al., 2021).

IS*6110* is often inserted in the environment of genes implied in “replication, recombination and repair,” “transcription,” “cell wall/membrane/envelope biogenesis,” and “coenzyme transport and metabolism pathways” GO categories according to the literature (Reyes et al., 2012). These four categories are also the most frequently identified in our analysis. In fact, 21% (78/369) of genes around IS*6110* insertion sites of multicopy strains are involved in the “replication, recombination and repair” GO category. This category with a determined function is the most frequent in our analysis. Genes encoding replication, recombination, and DNA repair functions seem to play an important role in the evolution of highly clonal bacteria such as *M. bovis* (Vultos et al., 2008; Mestre et al., 2011; Reis et al., 2021). Other frequent categories are “Intracellular trafficking, secretion, and vesicular transport” (7%, 26/369), “Cell wall/membrane/envelope biogenesis” (6%, 22/369), and “Transcription” (6%, 22/369) or “Cell motility” (5%, 20/369). IS*6110* insertions in or near these genes could play a role in *M. bovis* genome plasticity.

New IS*6110* insertion sites in *M. bovis* genomes have been described in this study, especially in SB0120-CO, SB0120-DHV, Cluster A/F4 family, and Cluster F/Eu2 clonal complexes such as insertion site 15 interrupting *cmr* gene or insertion site 24 interrupting *cyp144* gene. Some IS*6110* insertion sites have already been described in other MTBCs, suggesting that an ipl region could be common to several MTBC species. For example, the ancestral IS*6110* is present in almost all MTBC strains, and some other IS*6110* can be found in the CRISPR-Cas region (Hermans et al., 1990; Thorne et al., 2011; Refrégier et al., 2020). Moreover, the 3 insertion sites highlighted in our study that are common to several clusters have already been described as ipl in previous studies: the *plcD* region (insertion site 19) (Gordon et al., 1999; Sampson et al., 1999; Viana-Niero, 2006; Roychowdhury et al., 2015), insertion site 33 around *radD* (Chen et al., 2015), and insertion site 50 around IS*1547* (Fang et al., 1999) (Supplementary Table 2). The result gene regulation of IS*6110* insertion in these hot spots can be different depending on the IS*6110* orientations and/or insertion sites in the same genomic region and can lead to gene interruption or expression regulation (Sampson et al., 1999; Warren et al., 2000; Vera-Cabrera et al., 2001; Soto et al., 2004; Kim et al., 2010).

As expected, given that our strains are issued from well-established infected animals, IS*6110* insertion sites were not identified in genes previously described as essential for virulence (Sassetti and Rubin, 2003; Forrellad et al., 2013). However, some genes shown in our study as being interrupted by IS*6110*, such as *plcD*, were described as conferring an increased virulence for thoracic tuberculosis and a role in bacterial persistence within macrophages (Raynaud et al., 2002; Kong et al., 2005; Forrellad et al., 2013). Nonetheless, and in line with the fact that the strains in our study were infectious to animals from which they were isolated, other authors suggested that PLCs could play a less important role in bacterial virulence (Le Chevalier et al., 2015). However, genes such as *plcD* and *rpfD* have homologous genes, and their inactivation could be overcome by other homolog genes as reported by Kana and collaborators for *rpf* genes (Raynaud et al., 2002; Kana et al., 2008; Shafipour et al., 2021).

IS*6110* can also lead to gene regulation *via* its strong promoter as observed in the B strain on *phoP*. This insertion upstream of this essential gene for virulence probably led the strain to adapt to the human host and provoked an important multidrug-resistant (MDR) tuberculosis outbreak due to *M. bovis* (Soto et al., 2004). No such IS*6110* insertion leading to *phoP* regulation was found in our study. Some studies suggest that IS*6110* inserted no more than 400 bp upstream of a gene and in the same transcriptional orientation may upregulate its expression (Safi et al., 2004; Soto et al., 2004; Alonso et al., 2013). Such type of insertions has been detected in our analyses (data not shown). However, as they deserve complementary *in vivo* or *in vitro* studies with the corresponding strains, we cannot assess if they play any role on virulence or other survival traits.

## Conclusion

Contrary to what was expected from the literature, this study shows that 35% of the strains representative of the French diversity possess more than one IS*6110*. In addition, 10% that belong to a specific monophyletic clade of the Eu3 clonal complex present a high IS*6110* copy number. These multicopy strains are those circulating in French regions where bTB is most prevalent. IS*6110* insertion sites appear to be stable within specific genotypes over time and between host species, suggesting that IS*6110* transposition is not an evolutionary driver for modern French *M. bovis* strains at least over a 15-year period. Moreover, the correlation between the epidemiological success of *M. bovis* strains and multicopies of IS*6110* leads us to consider whether it could be the consequence of an increase in their fitness due to the genetic changes originated by IS*6110* transposition which could have occurred during ancient evolutionary diversification events. Any phenotypical consequence of IS*6110* insertions should need confirmation by *in vitro* or *in vivo* experiment.

## Data Availability Statement

The original contributions presented in the study are included in the article/Supplementary Material, further inquiries can be directed to the corresponding author.

## Author Contributions

LM, MLB, and FB supervised this study. CCh performed bioinformatics work and wrote the first draft. CCo provided bioinformatics support for data analysis. All authors contributed to this study correction, discussed the results and their interpretation, and approved the submitted version.

## Conflict of Interest

The authors declare that the research was conducted in the absence of any commercial or financial relationships that could be construed as a potential conflict of interest.

## Publisher’s Note

All claims expressed in this article are solely those of the authors and do not necessarily represent those of their affiliated organizations, or those of the publisher, the editors and the reviewers. Any product that may be evaluated in this article, or claim that may be made by its manufacturer, is not guaranteed or endorsed by the publisher.

## Footnotes

1
https://github.com/najoshi/sickle

2
https://github.com/jhawkey/IS_mapper
