# Supplementary figures and images for: IS6110 Copy Number in Multi-Host Mycobacterium bovis Strains Circulating in Bovine Tuberculosis Endemic French Regions
Source: Front Microbiol. 2022 Jun 23;13:891902. doi: 10.3389/fmicb.2022.891902 (PMC9260277; doi:10.3389/fmicb.2022.891902)

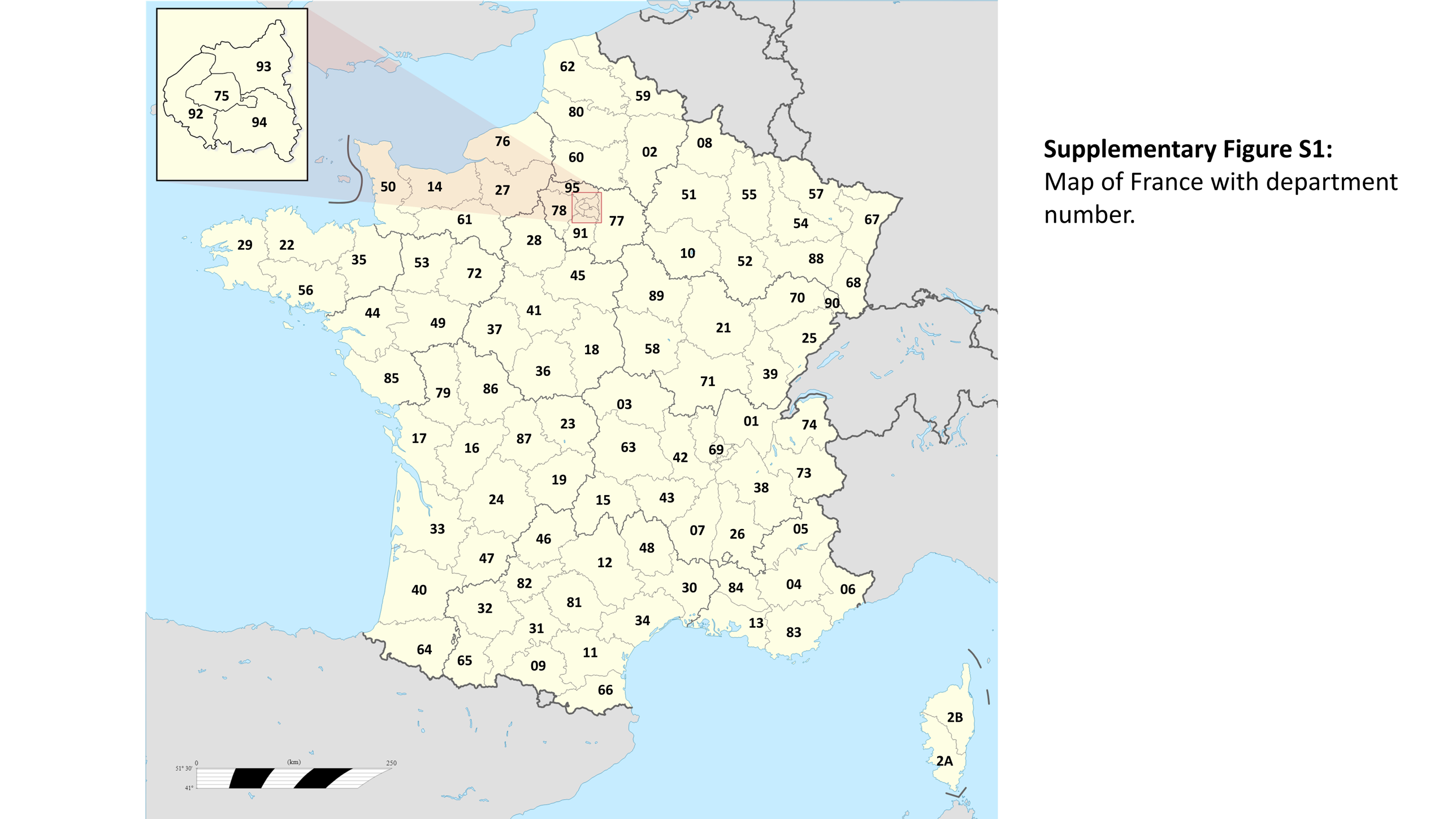

Supplement: Supplementary file 1 [file Image_1.JPEG]
