# Supplementary material for: IS6110 Copy Number in Multi-Host Mycobacterium bovis Strains Circulating in Bovine Tuberculosis Endemic French Regions
Source: Front Microbiol. 2022 Jun 23;13:891902. doi: 10.3389/fmicb.2022.891902 (PMC9260277; doi:10.3389/fmicb.2022.891902)
Supplement: Supplementary file 4 [file Table_3.DOCX]

| IS*6110* site | IS start | IS end |
| --- | --- | --- |
| 1 | 1525329 | 1523789 |
| 2 | 1898082 | 1896575 |
| 3 | 1978901 | 1980457 |
| 4 | 1997211 | 1995662 |
| 5 | 2162993 | 2162983 |
| 6 | 2213227 | 2212851 |
| 7 | 2252820 | 2254342 |
| 8 | 2260286 | 2260288 |
| 9 | 2262285 | 2260849 |
| 10 | 2660538 | 2662053 |
| 11 | 2711171 | 2710923 |
| 12 | 3088854 | 3087334 |
| 13 | 3091905 | 3093426 |
| 14 | 3095858 | 3095868 |
| 15 | 3521560 | 3521558 |
| 16 | 3681357 | 3682924 |
| 17 | 4127600 | 4126246 |
| 18 | 4239175 | 4239179 |

**Supplementary Table 3.** SB0120-CO nucleotide position of IS*6110* sites determined by ISMapper. IS start and end positions were identified in Mb3601 reference genome.
